# Editorial: Regulatory network in embryonic development and implantation

**DOI:** 10.3389/fphys.2024.1424967

**Published:** 2024-05-14

**Authors:** Yao Xu, Lin Zhao, Wenjing Wang

**Affiliations:** ^1^ Shanghai Key Laboratory of Maternal Fetal Medicine, Department of Assisted Reproduction, Clinical and Translational Research Center, Shanghai First Maternity and Infant Hospital, School of Medicine, Shanghai Institute of Maternal-Fetal Medicine and Gynecologic Oncology, Tongji University, Shanghai, China; ^2^ Department of Biomedical Engineering, Duke University, Durham, NC, United States; ^3^ Reproductive Medicine Center, Zhongshan Hospital, Fudan University, Shanghai, China

**Keywords:** early embryonic development, embryo implantation, endometrial aging, trophoblastic vesicles, fertility

“Where do I come from?” This is not only a philosophical issue raised by ancient Greek philosophers, but also a scientific question derived from the continuous development of human society. For thousands of years, humans have never stopped pursuing self-cognition and exploring the mysteries of life origins. In recent years, the continuous decline in global fertility rates has raised the issue of reproduction to an unprecedented level.

From the initiation of male and female gametogenesis to sperm-egg fusion which triggers the process of fertilization, the developmental process of a new life begins. The zygote undergoes several rounds of cleavage, differentiation, and migration. Eventually, it implants into the maternal uterus, completing the first intimate contact and interaction between the embryo and the maternal uterus. Undoubtedly, this process is of great significance for ensuring the ultimate successful pregnancy. During this period, the embryo experiences a maternal-to-zygote transition, where cells undergoes a series of rapid changes and dynamic events, all is strictly regulated by genetics, epigenetics, metabolism, and other factors. Any procedural errors or micro-environmental interferences during this process can lead to the reproductive diseases. Thus, investigating the molecular basis and mapping the regulating network of early embryonic development and implantation may enable us to monitor and elucidate the spatial-temporal dynamic changes of early life, which may ultimately deepen our understanding of the life’s origins. Moreover, such insights could also benefit us greatly in fertility guidance, clinical diagnosis, and treatment of reproductive diseases.

In recent years, the rapid development of multiple technologies has greatly accelerated our discoveries and explorations of phenotypes and mechanisms of early embryonic development. The comprehensive promotion and application of assisted reproductive technology (ART) in the field of infertility treatment, along with the high-resolution microscope combined with time-lapse imaging and artificial intelligence-assisted application, enable us to observe and manage the development process of pre-implantation embryos more efficiently and clearly. The iteration of high-throughput sequencers and high-resolution mass spectrometry makes the detection of nucleic acids and proteins in micro amounts of samples more accurate and reliable. These achievements have laid an important foundation for revealing the regulatory network, depicting the dynamic changes in cell lineage differentiation during embryonic development and implantation, and exploring the mechanisms of reproductive diseases.

This Research Topic aims to display novel findings or excellent ideas that digging into the key events during early embryonic development, uncover the regulatory networks in cell fate determination, and map the dynamic changes in maternal-fetal communication during implantation. In this issue, we present four articles, including three original research and one review article, with the intention of offering valuable support to both clinical and basic research in embryology and developmental biology.

ART is currently the most widely used technique for treating infertility in clinical practice, conventional treatments include artificial insemination, *in vitro* fertilization-embryo transplant (IVF-ET), intracytoplasmic sperm injection (ICSI), and Preimplantation genetic testing (PGT). ART has helped millions of families to have their own babies. However, there are still a considerable number of patients who cannot obtain fertility through ART, among which, early embryonic development arrest and fertilization failure are two of the most common disease phenotypes. In the first article, Zhang et al. collected the developmentally arrested embryos paired with control embryos, by performing high-throughput mRNA sequencing, they discovered 22 potential hub genes (highly connected genes) which might play critical roles in the early embryonic developmental process. ICSI is an effective treatment for severe male infertility and previous fertilization failure. However, recurrent unexpected fertilization failure or unexpected low fertilization continuously troubles clinicians and embryologists. Based on this, Xue et al. firstly performed a retrospective analysis of the Pb2 extrusion time and further confirmed that artificial rescue oocyte activation with calcium ionophore at 5 h after ICSI is a useful strategy for improving the oocyte fertilization rate. This study provides new clues for early rescue oocyte activation (EROA) to improve clinical fertilization rates.

Trophoblastic vesicles (TVs), as extra-embryonic tissues, can elongate *in vivo* even in the absence of embryonic tissues. Several studies have shown that TVs were generated from D12 bovine embryos elongated only *in vivo*, as well as being derived from D7 and D8 in vitro-produced blastocysts. However, TVs have not been produced at other stages, and molecular mechanism studies of these tissues have primarily focused on the trophectoderm. To address this gap, Degrelle et al. generated bovine TVs at D8, D12, and D15 and transferred them *in vivo* for 3, 6, or 9 days, respectively, to compare their elongation rates and molecular profiles at different times after the transfer. This study investigates the similarities and differences between the elongation rates and molecular profiles of TVs and those of the whole conceptuses, as well as patterns described for other models of elongation, providing insights for further exploration of embryo implantation and subsequent placenta formation.

In the last review article, Chemerinski et al. focused on the effect of maternal aging on reproduction and provided an overview of endometrial aging and its impact on pregnancy outcomes. The authors explored the mechanisms behind endometrial aging, including its cellular components, and reviewed human clinical studies to deepen our understanding of the contribution that the endometrium makes to age-related declines in fertility.

In summary, the articles collected in this Research Topic present novel advancements in mapping the regulatory network of early embryonic development and implantation. We are looking forward to having more excellent research to broaden our knowledge in the field of reproductive biology continuously.

